# A Monoclonal Antibody Based Capture ELISA for Botulinum Neurotoxin Serotype B: Toxin Detection in Food

**DOI:** 10.3390/toxins5112212

**Published:** 2013-11-18

**Authors:** Larry H. Stanker, Miles C. Scotcher, Luisa Cheng, Kathryn Ching, Jeffery McGarvey, David Hodge, Robert Hnasko

**Affiliations:** 1 Foodborne Toxin Detection and Prevention Unit, United States Department of Agriculture, Agricultural Research Service, 800 Buchanan Street, Albany, CA 94510, USA; E-Mails: Miles.Scotcher@dupont.com (M.C.S.); luisa.cheng@ars.usda.gov (L.C.); kathryn.ching@ars.usda.gov (K.C.); jeffery.mcgarvey@ars.usda.gov (J.M.); robert.hnasko@ars.usda.gov (R.H.); 2 DuPont Industrial Biosciences, Palo Alto, CA 94304, USA; 3 United States Department of Homeland Security, Washington, DC 20528, USA; E-Mail: David.Hodge@HQ.DHS.GOV

**Keywords:** monoclonal antibodies, botulinum neurotoxin serotype B, capture ELISA, toxin detection in food

## Abstract

Botulism is a serious foodborne neuroparalytic disease, caused by botulinum neurotoxin (BoNT), produced by the anaerobic bacterium *Clostridium botulinum*. Seven toxin serotypes (A – H) have been described. The majority of human cases of botulism are caused by serotypes A and B followed by E and F. We report here a group of serotype B specific monoclonal antibodies (mAbs) capable of binding toxin under physiological conditions. Thus, they serve as capture antibodies for a sandwich (capture) ELISA. The antibodies were generated using recombinant peptide fragments corresponding to the receptor-binding domain of the toxin heavy chain as immunogen. Their binding properties suggest that they bind a complex epitope with dissociation constants (KD’s) for individual antibodies ranging from 10 to 48 × 10^−11^ M. Assay performance for all possible combinations of capture-detector antibody pairs was evaluated and the antibody pair resulting in the lowest level of detection (L.O.D.), ~20 pg/mL was determined. Toxin was detected in spiked dairy samples with good recoveries at concentrations as low as 0.5 pg/mL and in ground beef samples at levels as low as 2 ng/g. Thus, the sandwich ELISA described here uses mAb for both the capture and detector antibodies (binding different epitopes on the toxin molecule) and readily detects toxin in those food samples tested.

## 1. Introduction

Foodborne botulism is a serious condition that is often fatal if untreated. The causative agent, botulinum neurotoxin (BoNT, EC 3.4.24.69) is produced by *Clostridium botulinum*. Seven serotypes of BoNT (A–G) have been described [1], A, B, E, and F are most frequently associated with human cases of botulism [2,3]. A total of 139 cases of Foodborne botulism were reported in the US between 2001 and 2007: 76 cases caused by intoxication of BoNT/A; 46 cases with BoNT/E; and 10 cases with BoNT/B. In the same time period, BoNT/B was responsible for 387 of the 663 cases of infant botulism reported by the CDC [4]. Foodborne botulism associated with serotype B is less common, however, the largest outbreaks occurred in the United States and the United Kingdom. There were 59 cases diagnosed in 1977 with type B botulism in Michigan, and 27 patients (one death) in 1989 with BoNT in the UK [5,6]. 

BoNTs are dichain protein toxins consisting of an ~100 kDa heavy chain (HC) and ~50 kDa light chain (LC) linked by a single disulfide bond. The HC functions by binding nerve cells and facilitates the internalization of the LC, a zinc metalloprotease, into pre-synaptic neurons at the neuromuscular junction [7,8]. The LC of BoNT/A cleaves synaptosomal-associated protein 25 (SNAP-25) whereas the LC of BoNT/B cleaves synaptobrevin-2 [9,10]. Either cleavage event prevents the docking of acetylcholine-carrying vesicles with the presynaptic membrane, effectively blocking the release of the neurotransmitter into the neuromuscular junction and ultimately prohibiting the contraction of the muscle [8].

We recently reported development of a sandwich ELISA for the detection of BoNT/A [11]. Using multiple mAbs, one for capture and others for detection, we demonstrated a limit of detection in chemiluminescent ELISA and in an electrochemical luminescent assay in the low pg/mL range and applied this assay to different food matrices [12]. Similar efforts with the B serotype of BoNT met with only partial success [13]. A series of mAbs specific for BoNT/B were identified that bound toxin in direct binding ELISA and on Western blots but these antibodies failed to bind toxin in solution. Modification of the screening assay to detect antibodies able to capture toxin under physiological conditions resulted in identification of a single mAb that was able to capture toxin. The resulting sandwich ELISA relied on a polyclonal antibody for the detector reagent. 

Here we report the development of additional BoNT/B mAbs capable of binding toxin in solution. Characterization of these antibodies, their binding specificity and affinity constants is reported. Development of a mAb/mAb capture ELISA capable of detecting BoNT/B in the low pg/mL range in buffer and in complex food matrices is presented. 

## 2. Materials and Methods

### 2.1. Reagents

Solutions at 1 mg·mL^−1^ of botulinum neurotoxin serotypes A–G and BoNT/A toxoid were purchased from Metabiologics Inc. (Madison, WI, USA). Bovine serum albumin (BSA), ovalbumin (OVA), goat anti-mouse immunoglobulin G conjugated to horseradish peroxidase (IgG-peroxidase) #A-4416, polyoxyethylene sorbitan monolaurate (Tween-20), Sigma Adjuvant System #6322, Protein-G conjugated Sepharose #P-32196, and the following buffers: 0.01 M phosphate buffered saline (PBS) #P-3813, 0.138 M NaCl, 0.0027 M KCl, pH 7.4; and 0.02 M TRIS-buffered saline (TBS) #T-5912, 0.9% NaCl, pH 7.4 were purchased from Sigma Chemical Co. (St. Louis, MO, USA). Black, Maxisorp 96-well Nunc microtiter plates were obtained from PGC Scientific (Gaithersburg, MD, USA), and SuperSignal Femto Max Sensitivity substrate was purchased from Pierce Inc. (Rockford, IL, USA). Non-fat dry milk (NFDM) and milk samples used in spiking experiments were purchased from a local food store. Luminescence was measured using a Perkin-Elmer Victor-3 microplate reader. Data were exported to Microsoft Excel for further analysis.

### 2.2. Recombinant BoNT/B-GST Fusion Proteins

**Figure 1:**
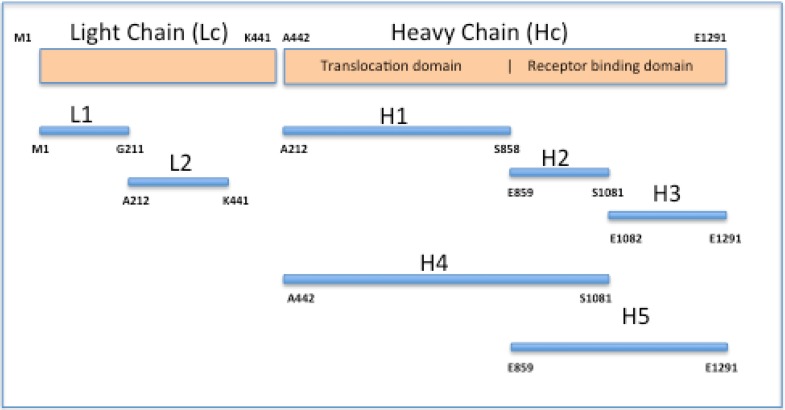
Schematic of recombinant-GST peptide fragments of BoNT/B light and heavy chains. Pepide fagmens were expressed as fusions to GST at the N-terminal.

Recombinant fragments of BoNT/B light and heavy chain (Lc1, Lc2, Hc1, Hc2, Hc3, Hc4, and Hc5) fused with glutathione transferase (recBoNT/B-GTS) were generated (see Figure 1), expressed in *Escherichia coli*, purified, and used to immunize mice as previously described [13]. These toxin fragments are referred to as recLc1-GST, recLc2-GST, recHc(1-5)-GST. The Institutional Biosafety Committee approved Recombinant DNA methods used in this study. DNA sequences of the mAbs described in this study have been deposited in GenBank and accession numbers are listed in Table 1.

### 2.3. Monoclonal Antibody Production and Screening Assay

Monoclonal antibody production was as previously described [11,13]. Briefly, recombinant peptide fragment recHc5-GST (265 µg/mL) was mixed with Sigma Adjuvant System according to manufacturer’s instructions (Sigma-Aldrich, St. Louis, MO, USA). Female BALB/cJ mice (Simonsen Laboratories, Gilroy, CA, USA) were immunized three times at 2-week intervals by intraperitoneal injection (i.p.) (100 µL). Following immunization with the recombinant recHc5-GST fragment, hybridomas were generated and supernatants from the cell fusion plates were evaluated for mAbs using a capture-capture ELISA screen. The Institutional Animal Care and Use Committee of the United States Department of Agriculture, Western Regional Research Center approved the experimental procedures used in these studies (protocol #04-1-H-05, 09-3). All animal experiments and husbandry involved in the studies presented in this manuscript were conducted under the guidelines of the U.S. Government Principles for the Utilization and Care of Vertebrate Animals Used in Testing, Research and Training.

### 2.4. Capture-Capture Screening ELISA

The capture-capture ELISA screening method used was previously described [13]. Briefly microtiter plates were coated with a 1 µg/mL solution of goat anti-mouse IgG Fc gamma #AP127 specific antibody (Millipore, Billerica, MA, USA) in 0.05 M sodium carbonate buffer, pH 9.6 overnight at 4 °C. Non reacted binding sites were blocked by adding 3% non-fat dry milk in Tris-buffered saline containing 0.05% Tween-20 (NFDM-TBST). Following a 1 h incubation at 37 °C culture supernatants from the fusion plates were added and the microtiter plates were incubated at 37 °C for 1 h. Plates were washed three times in TBST and a solution of BoNT/B in NFDM-TBST (200 ng/mL) was added and the plates were incubated at 37 °C for 1 h. Plates were washed three times as before, then a 1 µg/mL solution of anti-BoNT/B rabbit polyclonal antibodies (Metabiologics) in NFDM-TBST was added and the plates were incubated at 37 °C for 1 h. Plates were washed three times as before, then a 1 µg/mL solution of goat anti-rabbit HRP-conjugated polyclonal antibodies #A6154 (Sigma-Aldrich) was added and the plates were incubated at 37 °C for 1 h. Plates were again washed three times, and binding was visualized using SuperSignal West Dura Extended Duration Substrate (Pierce, Rockford, IL, USA) according to manufacturer’s instructions.

Cells from the wells giving positive signals for antibody production were cloned by limiting dilution, hybridomas expanded, and small amounts (usually less than 10 mL) of ascites fluids obtained (Covance Research Products, Inc., Denver, PA, USA). Antibodies were purified by affinity chromatography on Protein-G Sepharose. Bound antibody was eluted with 0.1 M glycine-HCl, pH 2.7 and dialyzed overnight *versus* PBS. Protein concentrations were determined with a BCA-kit (Pierce) using the microplate method suggested by the manufacturer. The isotype of each antibody was determined using the SBA Clonotyping System/HRP in ELISA format, according to manufacturer’s instructions (Southern Biotech, Birmingham, AL, USA). Western blots were as described [11]

### 2.5. Capture ELISA

The capture ELISA previously described [11] was used with the following modifications. Microtiter plates were coated with capture antibody, 100 µL of a 0.5 µg/mL solution of mAb in 0.05 M sodium carbonate buffer, pH 9.6 overnight at 4 °C. Non reacted binding sites were blocked by adding 3% non-fat dry milk in TBST (NFDM-TBST). Toxin standards in TBST, or spiked samples were added (100 µL/well) and the plates incubated at 37 °C for 1 h. Next, detector antibody (each mAb was labeled with biotin [11] was added (100 µL at 5 µg/mL in NFDM-TBST), the plates incubated for 1 h at 37 °C, washed. One hundred µL of a 1/20,000 dilution of streptavidin-HRP (Zymed, S. San Francisco, CA, USA) was added, the plates incubated 1 h at 37 °C and visualized using luminescent substrate (SuperSignal ELISA Femto, Pierce #37074) according to manufacturer’s instructions. Luminescence was measured on a Victor 3 plate reader.

### 2.6. Antibody-Antigen Binding Affinity Measurements

Real time binding assays between purified antibodies and purified BoNT/B protein were performed using biolayer interferometry with an Octet QK system (Forte-bio, Menlo Park, CA, USA). The system measures light interference on the surface of a fiber optic sensor, which is directly proportional to the thickness of molecules bound to the surface. Binding of a partner molecule to the tethered target results in thickening of the surface, which is monitored in real time. In this study, mAbs were bound to kinetics grade anti-mouse IgG Fc Capture Biosensors (Forte-bio) at 5 µg/mL in PBS for 3600 s. Unbound antibodies were removed from the surface of the sensors by incubation in PBS (300 s). Probes coupled to antibody were allowed to bind to BoNT/B at 10, 1.0, and 0.1 nM for 3600 s. Binding kinetics were calculated using the Octet QK software package (Data Acquisition 7.0), which fit the observed binding curves to a 1:1 binding model to calculate the association rate constants. The BoNT/B was allowed to dissociate by incubation of the sensors in PBS for 1800 s. Dissociation kinetics were calculated using the Octet QK software package, which fit the observed dissociation curves to a 1:1 model to calculate the dissociation rate constants. Equilibrium dissociation constants were calculated as the kinetic dissociation rate constant divided by the kinetic association rate constant. 

#### Food Analyses

Food samples, fat free milk, 2% fat milk, whole milk, 73% and 92% lean ground beef, were obtained from local grocery stores, spiked with BoNT/B, and analyzed by capture ELISA. All samples were spiked as follows. Milk samples (1 mL) were spiked by addition of 10 µL of toxin at the level indicated. Non-spiked control samples also were prepared and analyzed. Following addition of toxin the samples were incubated at room temperature for 30 min, centrifuged at 1000 × g for 5 min at 4 °C using a microcentrifuge. An aliquot was then recovered, below the fat layer, diluted 1:1 with TBST, and analyzed by capture ELISA, see above. For ground beef sample, 1 g of sample was placed into a small zip lock plastic bag, spiked, and incubated for 30 min. TBST (2 mL) was then added to the bag and the sample was stomached by hand (30–60 s). A second 2 mL of TBST was added to the sample, the bag tilted and the liquid removed and centrifuged at 1000 × g for 5 min at 20 °C. The supernatant was recovered and diluted 1:1 with TBST and analyzed by the capture ELISA described above.

## 3. Results

### 3.1. Isolation and Characterization of Monoclonal Antibodies

Earlier studies aimed at development of a sensitive capture ELISA for BoNT/B using mAbs for both the capture and detection reagents meet with only partial success [13]. Using a standard ELISA with immobilized antigen for screening cell fusion experiments, a number of mAbs were isolated. However, none of the antibodies so produced effectively bond toxin in solution under physiological conditions, and thereby were not useful as a capture antibodies in a sandwich ELISA. Furthermore, none of the mAbs produced in this earlier study were able to function as a detector antibody (even though they bound toxin in ELISA and on Western blots [13]. A single mAb able to capture toxin from solution eventually was identified but only after incorporating a double-capture ELISA screen [13]. In the studies described here the double-capture ELISA screen was used in an effort to identify additional capture and detector antibodies and useful antibody pairs for measurement of BoNT/B. 

**Figure 2:**
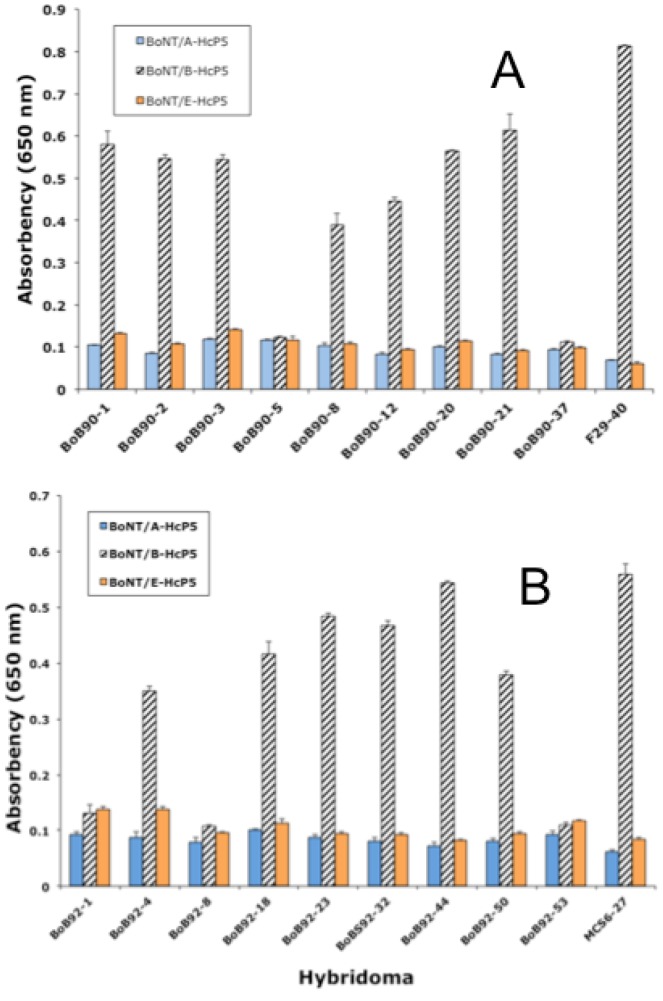
Antibody binding to recombinant BoNT-GST fusion proteins representing the receptor binding domain of the toxin HC for serotypes A, B, and E. Hybridoma supernatants analyzed by ELISA using recombinant peptides as antigen.

Following cell fusions, putative capture mAbs were trapped on microassay plates pre-coated with anti-mouse Ig (Fc) specific antiserum (see methods). We identified positive responses in 18 of ~2000 fusion wells screened. Wells were considered positive if the measured activity was at least 2–3 times the average response of all wells on the plate. Incorporating authentic BoNT/B into the screen (see methods) eliminates isolation of peptide specific mAbs in favor of those mAbs capable of binding the intact toxin molecule. It was hoped that this approach would guarantee that all of the mAbs detected in the screen were capable of capturing BoNT/B in solution. Equally important, the capture-capture screen does not detect the vast majority of monoclonal antibodies that are detected in a traditional ELISA screen but fail to capture antigen in solution. None of the 18 mAbs identified in the initial screen bound toxin immobilized on microassay plates by ELISA. However, analysis of antibody binding to microtiter plate immobilized recGST-HcP5 peptide (the immunogen) demonstrated that 13 of the 18 mAbs bound the recGST-HcP5 peptide by ELISA, but only to the recGST-HcP5 peptide corresponding to the amino acid sequence found in toxin serotype B1, but not the H5 fragments based on the A1 or E1 serotypes (Figure 2).

**Figure 3:**
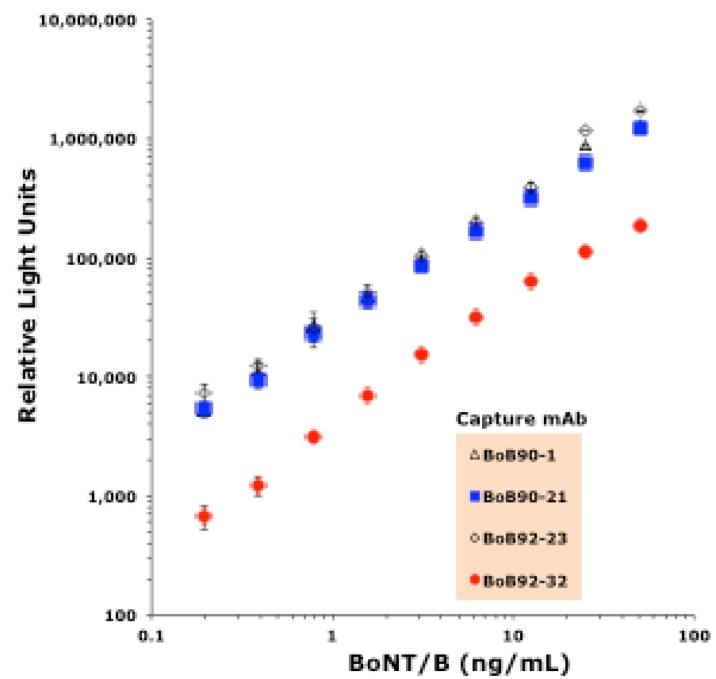
Capture-Capture sandwich ELISA. Identification of capture mAbs immobilized on anti-mouse Ig coated microtiter plates. Detection of bound toxin using a polyclonal anti-BoNT/B antiserum with mAb capture antibodies indicated. Data represents average of three replicates, error bars = ±standard deviation.

Four of the mAbs, BoB90-1, BoB90-21, BoB92-23, and BoB92-32, survived multiple rounds of cloning by limited dilution and were further analyzed. All four were able to function at capture antibody in a sandwich ELISA using a polyclonal anti-BoNT/B serum as detector (Figure 3). Subsequent ELISA titration experiments using BoNT/B immobilized on microtiter plates revealed that none of the isolates were able to bind toxin after it was adsorbed onto microtiter plates in a typical ELISA.

**Figure 4:**
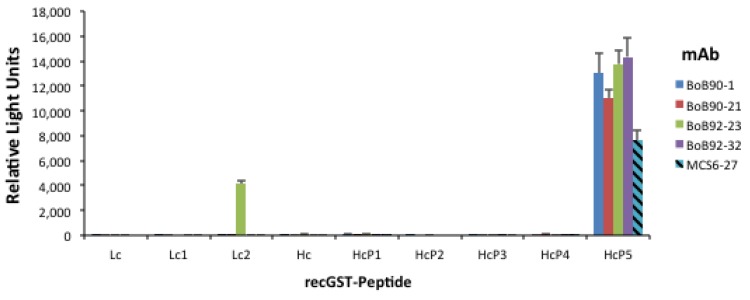
Monoclonal antibody binding to recombinant peptides of BoNT/B (see Figure 1). Antibodies MCS6-27 and F27-33 described earlier [13].

In an effort to further characterize the binding epitopes each mAbs was analyzed for binding to the recGST-peptide panel: LcP1; LcP2; HcP1; HcP2; HcP3; HcP4; and HcP5 peptides (Figure 1), immobilized on 96-well microtiter plates. Binding was observed to HcP5 peptide but not to the HcP2, HcP3, or HcP4 peptides suggesting that the epitope was destroyed when the HcP5 fragment was subdivided (Figure 4). In addition, mAb BoB92-23 demonstrated weak but consistently binding to the LcP2 peptide. 

Bilayer interferometry was used to measure the affinity constant (K_D_) of each of the four mAbs described here (Table 1). BoB92-23 showed the strongest binding, with a dissociation constant of 10.2 × 10^−11^ M. Weaker affinity constants were measured for the other mAbs. Isotype analysis indicated that BoB92-23 is an IgG2b while the remaining three mAbs are IgG1, all having kappa light chains (Table 1).

**Table 1:** Characteristics of BoNT/B monoclonal antibodies.

Antibody	Isotype	K_D_ (×10^−11^ M)
BoB90-1	IgG1, kappa	23.0
BoB90-21	IgG1, kappa	48.0
BoB92-23	IgG1, kappa	10.2
BoB92-32	IgG2b, kappa	48.3
MCS6-27	IgG1, kappa	8.6

**Figure 5:**
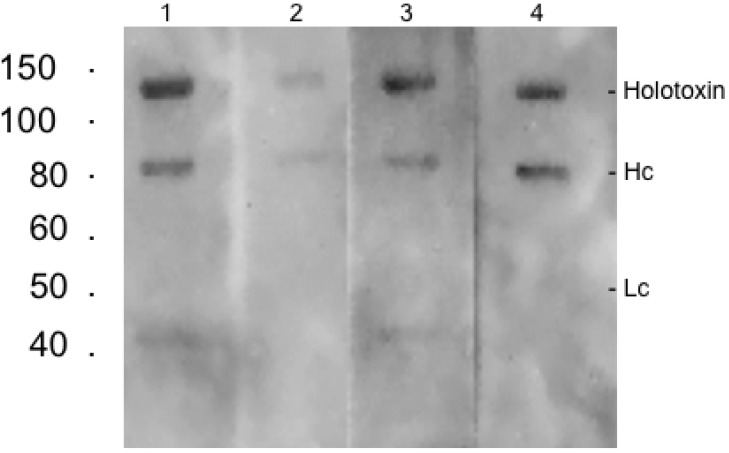
Western blot analysis of anti-botulinum serotype B mAbs. Lane 1, BoB90-1; Lane 2, BoB90-21; Lane 3, BoB92-23; Lane 4, BoB92-32. Each lane contained BoNT/B at 10 µg/mL plus DTT and probed with 10 µg/mL antibody. All lanes exposed for 10 min. Standards on right side represent Kilo Daltons.

Weak antibody binding to the 50 kDa toxin Hc and to the 150 kDa non-reduced holotoxin was observed on Western blots (Figure 5) following electrophoresis in the presence of DTT. The weak binding on Western blots was expected, since antibody binding to microtiter plate immobilized toxin by ELISA was minimal. The long exposure times needed to visualize bands in the Western blots suggests that heating toxin in SDS-PAGE sample buffer causes partial disruption of the antibody binding epitopes. Clearly SDS-PAGE modifies proteins, altering their surface charge and potentially denaturing tertiary structures. Changes of surface charge and/or structural changes that can eliminate binding epitopes also can occur following adsorption to a solid phase [14]. 

**Figure 6:**
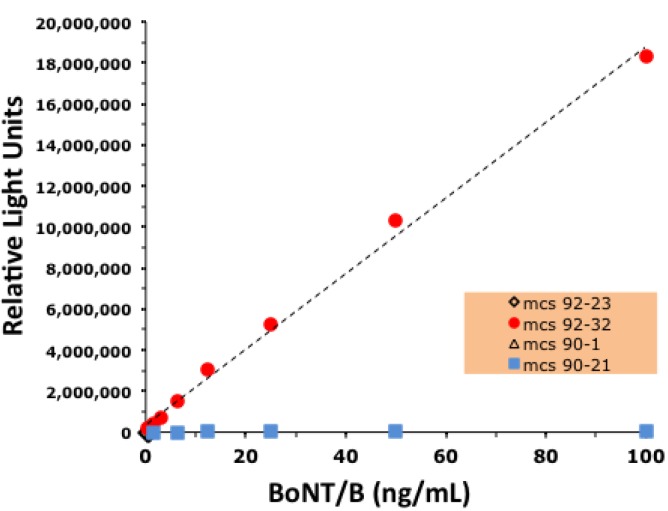
BoNT/B detection in a capture ELISA using mAb MCS6-27 as capture antibody and the four mAbs described here as detector antibodies. Points for MCS 92-23 and 90-1 hidden by the points for MCS 90-21. Line represents a linear curve fit, *R*^2^ = 0.996.

The ability of the four mAbs (mAbs BoB90-1, BoB90-21, BoB92-23, and BoB92-32) to function as detector antibodies using a previously produced capture mAb (MCS6-27) (Scotcher *et al*. 2010) was evaluated. These experiments, summarized in Figure 6, revealed that only one of the antibodies, biotin-labeled BoB92-32 functioned as a detector antibody. 

### 3.2. Capture ELISA

**Figure 7:**
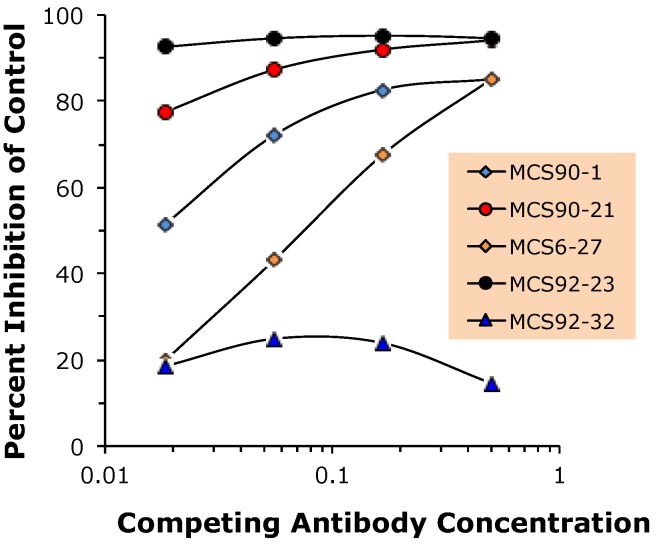
Competition capture -ELISA using mAb MCS6-27 as capture antibody. Toxin mixed with increasing concentrations of competing mAb (indicated on figure) and immediately added to the capture antibody coated well. Toxin detection using a polyclonal anti-BoNT/B.

These data are consistent with competition ELISA experiments (Figure 7) demonstrating that mAb BoB92-32 did not compete with mAb MCS6-27 for toxin binding at any of the concentrations tested. In contrast, mAbs BoB90-1, BoB90-21, and BoB92-23 competed with MCS6-27 for toxin binding suggesting that they bind the same or closely related epitopes. 

**Figure 8:**
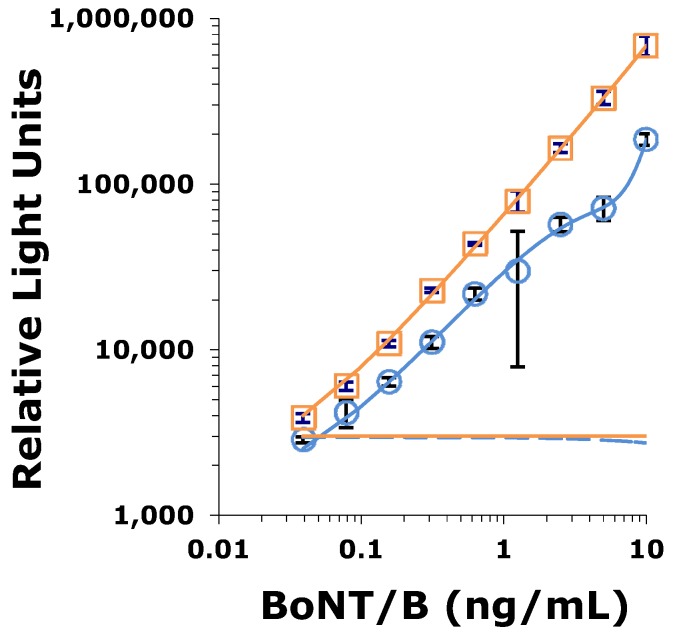
Capture ELISA comparing assay performance using either MCS6-27 (open blue circle) or BoB92-23 (open orange square) as capture antibody and BoB92-32 as detector antibody. Bars represent standard deviation (N = 3). Data fitted using 4-paramater-curve fitting. The average of the zero toxin control plus three standard deviations represented by the horizontal lines for BoB92-21 (solid) and MCS6-27 (dashed).

All possible combinations of the four mAbs described here plus mAb MCS6-27 were evaluated for development of a sandwich ELISA. These experiments (data not shown) demonstrated that only two capture/detector antibody pairs (BoB92-23/BoB92-32 and MCS6-27/BoB92-32) were useful for development of a capture ELISA. Careful titration experiments using BoNT/B holotoxin suggest that the combination BoB92-23 (capture) and BoB92-32 (detector) resulted in a more sensitive assay than pairing MCS6-27 (capture) with BoB92-32 (detector) (Figure 8).

**Figure 9:**
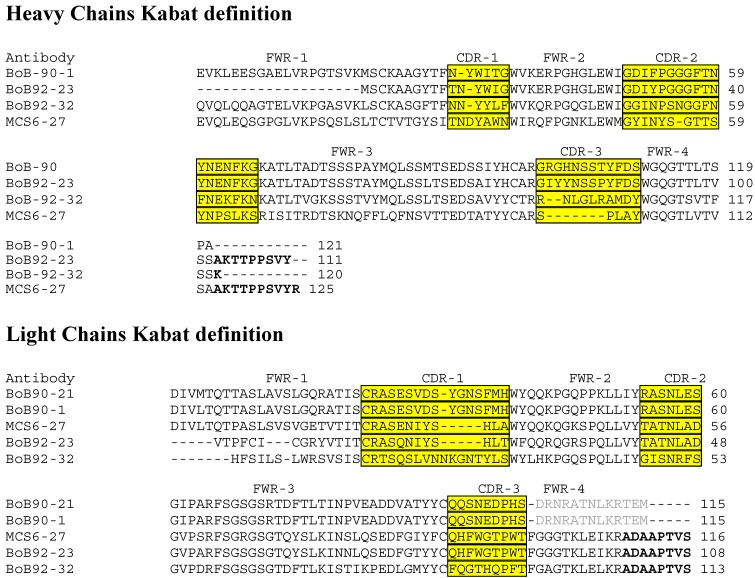
Clustal W2 alignment of the amino acid sequence deduced from the cDNA of the variable regions of the anti-BoNT/B monoclonal antibodies. The Framework Regions (FWR) and Complementarity Determining Regions (CD-1, -2, and -3) are indicated (boxed), bold text indicates constant region sequences.

In an effort to determine if the four mAbs represented separate fusion products of a clonally expanded lymphocyte population, the amino acid sequences of the light and heavy chain variable regions were determined. Inspection of the translated amino acid sequences of the combining region of each antibody (Figure 9) suggests that mAbs BoB92-23 and BoB92-31 are unique antibodies. MAb BoB90-1 and BoB90-21 have identical Lc sequences, but sequence information for the Hc of BoB90-21 was not obtained so they could represent independent hybridomas resulting from fusion of a clonally expanded lymphocyte population. The amino acid sequences of the variable region of the four antibodies described here are different from that of mAb MCS6-27 shown for convenience.

### 3.3. Food Analysis

**Supplemental Figure 1:**
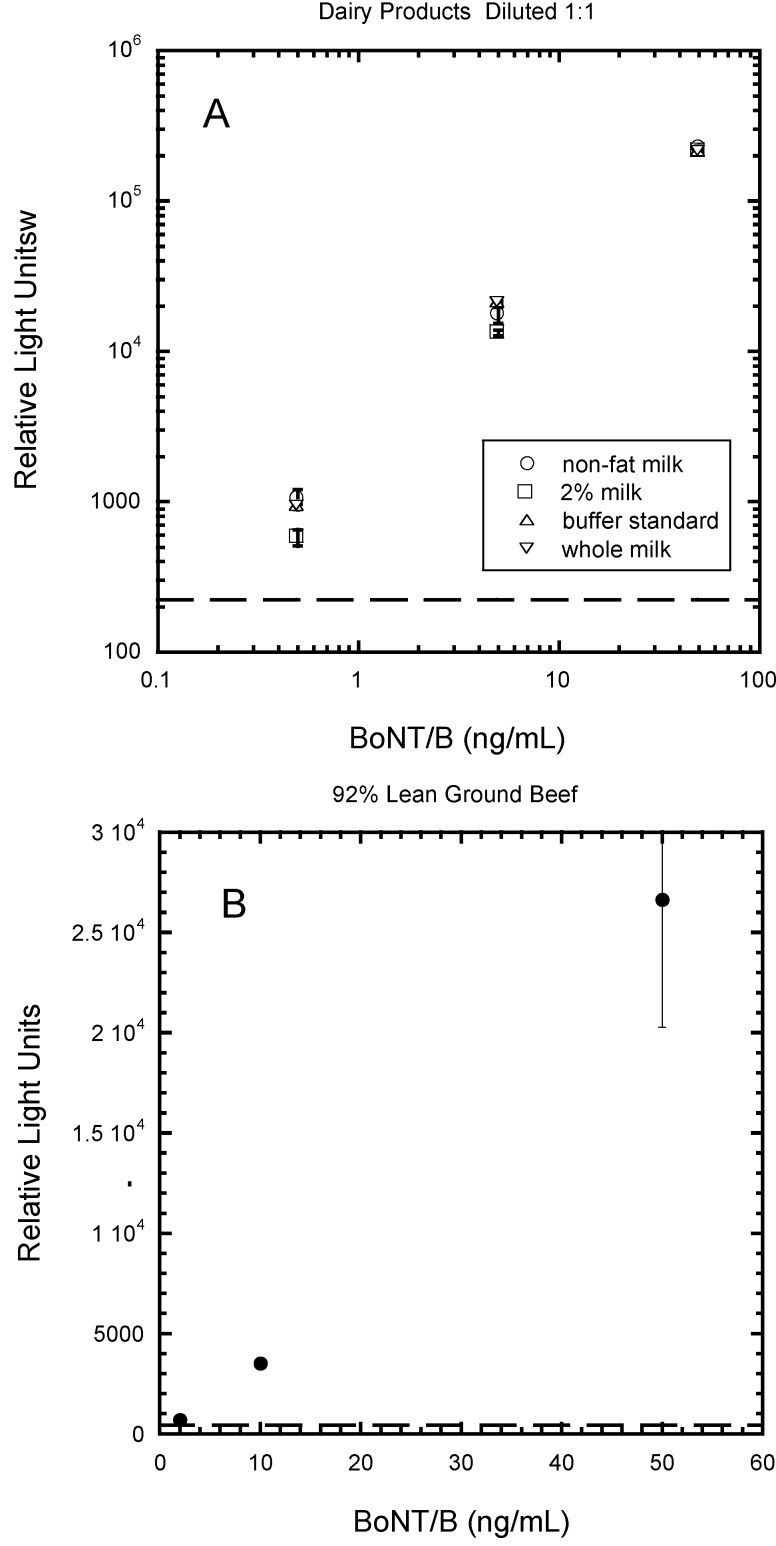
(**A**, **B**). Detection of BoNT/B spiked into food by capture ELISA.

The assay was next applied for evaluation of foods fortified at different concentrations with BoNT/B. In these experiments, all samples were fortified with BoNT/B at a constant volume (10 µL) of toxin and the samples were incubated at room temperature for 30 min before processing. These data (Supplemental Figure 1) were used to establish the L.O.D. (average of the buffer control plus three standard deviations). In all cases, the signal observed at the lowest spike level was higher than the L.O.D. Thus, the percentage toxin recovery (Table 2) could be calculated for each toxin concentration. In those dairy products tested, the percentage recovery varied from 63.8% to 131%. However, at the 50 and 5.0 ng/mL spike levels the recoveries were close to 100% (only one sample was below 89%). Greater variations of the percentage recovery were observed following analysis of the ground beef sample.

**Table 2:** Percentage recovery of BoNT/B spiked into different food matrices.

Percentage Recovery					
Matrix/Spike Level	50 ng/mL	10 ng/mL	5 ng/mL	2 ng/mL	0.5 ng/mL
Non Fat Milk	108 (54)^1^	nd^2^	89 (4.45)	nd	131 (0.655)
2% Milk	97 (48.5)	nd	76 (3.35)		73 (0.365)
Whole Milk	101 (50.5)	nd	105 (5.25)	nd	116 (0.58)
Ground Beef 95% Lean	63.8 (31.9)	116 (5.8)	nd	100 (2)^3^	nd

Notes: 1. Numbers in brackets refer to toxin level measured at each spike concentration using a buffer standard curve. Percent recovery = measured concentration/spike concentration × 100; 2. nd = not done; 3. The signal from beef sample spiked at 2 ng/mL was just at the L.O.D. for standard.

## 4. Discussion

We have previously used recombinant GST-fusion peptides of BoNT/B fragments for the successful generation of mAbs that bind intact neurotoxin. While these studies demonstrated the feasibility of using recombinant peptides of BoNT/B as immunogens, all of the resulting mAbs, except one, were unable to bind toxin in solution or act as a capture or detector antibody in a sandwich ELISA [13]. In these earlier experiments screening was based upon a traditional ELISA, *i.e.*, BoNT/B was immobilized in microtiter plates by absorption and hybridoma supernatants evaluated for mAb binding. Proteins absorbed to plastic surfaces can undergo structural modifications that can hide or destroy antibody-binding epitopes [14]. By modifying the screening strategy to a capture-capture assay one mAb (MCS6-27) that could be utilized as a capture antibody was identified (Scotcher *et al*. 2010). Likewise, none of the antibodies isolated from these earlier fusions functioned as detector antibodies. Thus, our initial goal of generating a mAb-based sensitive capture ELISA for BoTN/B was only partially meet. To identify additional capture and detector antibodies the capture-capture screen described here was incorporated to screen cell fusion experiments.

The poor binding observed following toxin adsorption to microassay plates and on Western blots following SDS-PAGE suggests that the binding epitope, localized to the receptor binding domain of the toxin Hc, is disrupted or masked by these treatments and each mAb is binding a conformational epitope. This is consistent with antibody binding to only the recombinant peptide corresponding to the entire receptor-binding domain of the toxin but not peptides H2 and H3 that divide the receptor-binding domain. 

The four new capture mAbs described here have epitopes located in the receptor-binding domain of BoNT/B Hc. Sequence analysis of the combining regions suggests that these antibodies are unique and different from our earlier developed capture mAb MCS6-27. However, competition ELISA experiments demonstrate that BoB90-1, BoB90-21, and BoB92-21 compete with mAb6-27 for toxin binding. In fact, BoB92-21 has a substantially stronger competition curve than seen when MCS6-27 competes with itself for toxin binding. These data and the assay performance observed in a sandwich ELISA using MCS6-27 *vs.* BoB92-21 as capture antibody suggest that BoB92-21 as capture antibody results in lower limits of detection.

Preliminary results indicate that a prophylactic, intravenous (iv) injection of µg amounts of BoB90-1, BoB90-21, BoB92-23, and BoB92-32 per mouse protected 100% of the mice studied from death or any symptoms of intoxication from an iv injection of 460 pg (100 mouse iv LD_50_ doses) of BoNT/B, consistent with the observation that these mAbs could bind BoNT/B *in vitro* under physiological conditions [15]. Research to more clearly define the parameters controlling the ability of these antibodies to neutralize toxin and rescue animals following exposure is the subject of future studies. However, these observations are consistent with earlier studies [13] suggesting that antibodies that failed to bind BoNT/B in physiological buffer did not bind toxin *in vivo* and failed to protect mice from the neurotoxic effects of BoNT/B. The ability of a mAb to capture antigen from solution appears to be an indicator for toxin neutralization potential.

Using our previously developed mAb MCS6-27 as the capture antibody, only mAb BoB92-32 was able to function as a detector antibody (Figure 6). These data suggest that the other three mAbs bind an epitope that is not available when toxin is first bound by mAb MCS6-27, or that BoB90-1, BoB90-21, and BoB92-23 bind the same or a closely related epitope.

The ability of BoB92-32 to act as a detector antibody is consistent with its failure to compete with MCS6-27 for toxin binding. Of the remaining three mAbs, BoB92-21 showed the strongest competition, greater than competing MCS6-27 with itself. The exact nature of this competition is not clear. The DNA sequences of the combining regions of these antibodies suggest that each represents a uniquely derived monoclonal antibody. 

Evaluation of all possible capture/detector combinations demonstrated that the best performance of a sandwich ELISA was observed using either BoB92-23 or MCS6-27 as capture antibody coupled with BoB92-32 as detector antibody. The combination BoB92-23/BoB92-32 showed slightly better performance than MCS6/27/BoB92-32 (Figure 8 consistent with the competition ELISA experiments. Using this antibody pair the L.O.D. was determined to be approximately 10–20 pg/mL. The mouse LD_50_ of the BoNT/B preparations used in our laboratory, when injected intraperitoneally was found to be ~10 pg/mL [15]. The sandwich ELISA described here has a comparable L.O.D. as the mouse bioassay (MBA) but requires only a few hours to complete *versus* 2–4 days. Analysis of spiked dairy products and ground beef at 50–0.5 ng/mL suggest that acceptable sample recoveries are obtained with the assay and that the sandwich ELISA represents a useful tool for rapid analysis of food for BoNT/B.

In summary, we have developed a sensitive sandwich ELISA specific for BoNT/B that is both rapid and easy to use. This assay is based on monoclonal antibodies for both the capture and detector reagents and thus represents an assay that can be consistently produced over time without the use of animals and the variability inherent in polyclonal antisera. We are currently exploring other labels and assay formats, including a lateral flow immunoassay able to detect and distinguish BoNT/A and BoNT/B in a few minutes [16].
